# Simultaneous silence organizes structured higher-order interactions in neural populations

**DOI:** 10.1038/srep09821

**Published:** 2015-04-28

**Authors:** Hideaki Shimazaki, Kolia Sadeghi, Tomoe Ishikawa, Yuji Ikegaya, Taro Toyoizumi

**Affiliations:** 1 RIKEN Brain Science Institute, 2-1 Hirosawa, Wako-shi, Saitama 351-0198, Japan; 2 Commonwealth Computer Research Inc., 1422 Sachem Pl., Unit #1, Charlottesville, VA 22901, U.S.A.; 3 Graduate School of Pharmaceutical Sciences, The University of Tokyo, 7-3-1 Hongo, Bunkyo-ku, Tokyo 113-0033, Japan; 4 Heart to Heart Science, Center for Information and Neural Networks, 1-4 Yamadaoka, Suita, Osaka, 565-0871, Japan; 5 Department of Computational Intelligence and Systems Science, Tokyo Institute of Technology, Yokohama 226-8502, Japan

## Abstract

Activity patterns of neural population are constrained by underlying biological mechanisms. These patterns are characterized not only by individual activity rates and pairwise correlations but also by statistical dependencies among groups of neurons larger than two, known as higher-order interactions (HOIs). While HOIs are ubiquitous in neural activity, primary characteristics of HOIs remain unknown. Here, we report that simultaneous silence (SS) of neurons concisely summarizes neural HOIs. Spontaneously active neurons in cultured hippocampal slices express SS that is more frequent than predicted by their individual activity rates and pairwise correlations. The SS explains structured HOIs seen in the data, namely, alternating signs at successive interaction orders. Inhibitory neurons are necessary to maintain significant SS. The structured HOIs predicted by SS were observed in a simple neural population model characterized by spiking nonlinearity and correlated input. These results suggest that SS is a ubiquitous feature of HOIs that constrain neural activity patterns and can influence information processing.

Information in the brain is represented by the collective spiking activity of multiple neurons^1^. Activity patterns of observed neurons are highly structured due to various underlying biological mechanisms including direct anatomical connections^2^,^3^, indirect connections mediated by unobserved neurons^4^,^5^, and intrinsic nonlinearity of individual neurons^6^,^7^. However, exploration of this structure is non-trivial due to limited data size in comparison to possible combinations of activity patterns that grow exponentially with population size.

To infer the structure of neural activity patterns from limited amount of data, the maximum entropy principle has been successfully applied^8^,^9^. Under this principle, the probability distribution of activity patterns is estimated to be the least structured distribution that is consistent with a set of observed activity statistics. Conventionally this maximum entropy distribution is statistically characterized by parameters of different orders, where the orders refer to the numbers of subset neurons that these parameters constrain. The model with the first-order parameters fits to the observed activity rates of individual neurons. The model that additionally includes the second-order parameters further adjusts the observed deviations of pairwise correlations from the chance coincidence expected from the individual activity rates. The second-order parameters are referred to as pairwise interactions. More generally, the model that includes up to the *k*-th (*k* = 3, 4, . . . ) order interactions adjusts simultaneous activation rates of *k* neurons from the expectation based on interactions up to the (*k*-1)-th order. Interactions beyond the pairwise interactions (*k*> 2) are collectively termed higher-order interactions (HOIs)^10^,^11^. Notably, these interactions refer to statistical dependency of neurons, and do not necessarily involve anatomical connections.

In earlier studies, individual activity rates and pairwise correlations alone could explain ~ 90% of variability in activity patterns of small populations of retinal ganglion cells^8^,^9^ and cortical neurons^12^,^13^. However, this does not exclude the existence of HOIs or limit their contribution to information processing. Indeed, the addition of HOIs to a statistical model significantly improved the goodness-of-fit to neural activities obtained from multi unit activity^14^,^15^, single unit activity^5^,^16^,^17^,^18^,^19^,^20^, and local field potential^21^,^22^ in both *in vivo* and *in vitro* preparations. Furthermore, HOIs are relevant in neural information coding^14^,^16^,^18^,^23^. However, previous studies have not identified a key feature in HOIs that summarizes the principal role of seemingly diverse HOIs.

One of the most striking features of neural population activity is simultaneous silence (SS). The spiking activity of individual neurons is known to be sparse^24^. As a result, the most commonly observed activity pattern in typical networks is the pattern in which all neurons are silent. Does SS involve HOIs? Indeed, departures from the level of expected SS from individual activity rates and pairwise correlations (excess SS) were empirically reported previously^16^,^17^,^18^,^19^,^20^. However, the significance of SS in characterizing HOIs of the population activity is not well understood.

Here, we examine SS in population activity of the hippocampal CA3 networks in cultured slices. Previous studies demonstrated that CA3 pyramidal cells in the organotypic slice cultures are wired with an in vivo-like connection probability of 15–30%^3^, and their spontaneous spike rates are closer to those of in vivo hippocampal neurons^25^, compared to neurons in acute slice preparations. We demonstrate that most local groups of hippocampal neurons that possess HOIs express excess SS. A single parameter that quantifies SS accounts for about 20% of the variability in population activity patterns that is produced by numerous HOIs. We then confirm specific oscillatory structure of HOIs at successive interaction orders predicted from the SS. Through modeling, we also demonstrate that correlated population activity caused by spiking nonlinearity and correlated input exhibits the same structure of HOIs, and that this structure conveys information of input. These results suggest that neurons are operating in a unique regime where they are constrained to be silent simultaneously.

## Results

### Simultaneous silence and HOIs of hippocampal neurons

**Figure 1:**
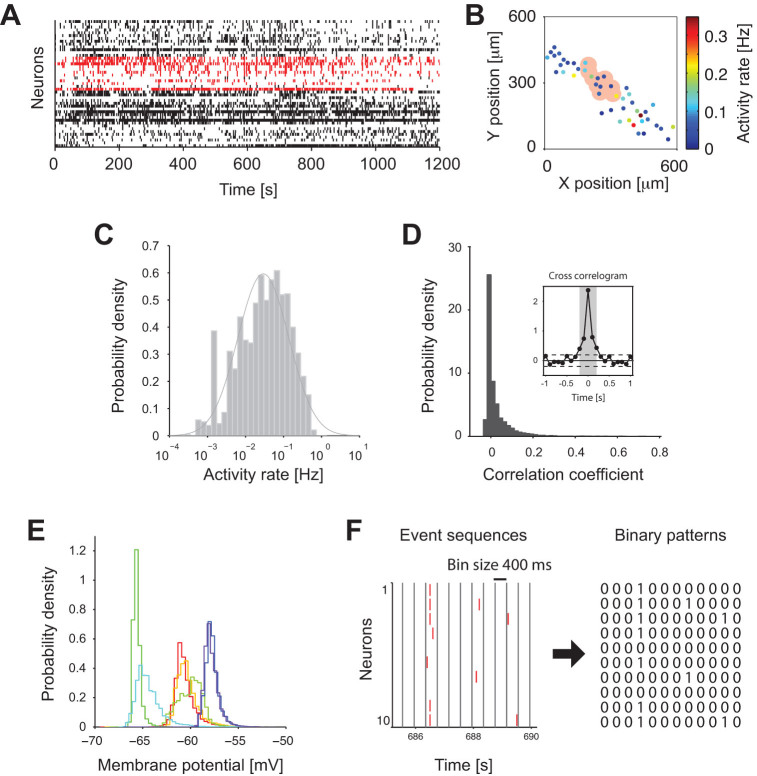
Ensemble activity of CA3 putative neurons detected by Calcium imaging. (A) Ensemble activity of 45 neurons from a single hippocampal slice. Small vertical ticks indicate *events* detected from calcium imaging signals. Ensemble activity of an example group of 10 neurons is marked in red. (B) Spatial distribution of neurons in the CA3 area of the slice in A. Each filled circle represents a position of a neuron. The color indicates activity rate of each neuron. The pink area corresponds to the example group highlighted in A. (C) Distribution of activity rates from neurons in all 20 slices. Solid line is a fitted log-normal distribution. (D) Distribution of correlation coefficients calculated from the event sequences (within a 100 ms window) from all the pairs of neurons in 1000 neighboring groups from 20 slices. The inset shows an average cross-correlogram from all the pairs of neurons. Dashed lines indicate ± 2 SD of the correlogram at 1–2 sec lags. The gray shading (−0.2 ms to + 0.2 ms) indicates the interval where the correlgoram exceeded the dashed lines. (E) Distributions of membrane potentials recorded from neurons (*n* = 7) in hippocampal slice cultures under the same condition as described in Methods. Different colors indicate different neurons. In all cases, the densities of the membrane potentials were characterized by a unimodal profile. (F) Construction of binary patterns from event sequences. The event sequences are binned using a window of 400 ms. In each bin, we denote ‘0’ if there is no event, and ‘1’ if there is at least one event.

We analyzed the spontaneous spiking activity of putative neurons in the hippocampal CA3 area of organotypic slice cultures, measured by the Calcium imaging method. Slices were prepared from postnatal day 7, and then cultivated from day 7 to 14 (see Methods). Neuronal activity was detected by onsets of calcium transients^3^,^26^,^27^,^28^, which provided event-timing data with a resolution of 100 ms. Fig. 1A and B display an example of population event activity of a single slice culture, and spatial positions and activity rates of individual neurons. We analyzed 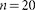 slices in total, and found the following features. First, activity rates of the neurons from all slices were distributed close to a log-normal distribution (Fig. 1C), similarly to spike rates of *in vivo* hippocampal CA3 neurons of awake rodents^29^,^30^. The rates of calcium events in individual cells computed from 2122 neurons in 20 slices were 0.073 ± 0.097 (mean ± standard deviation (SD) events/s; median 0.035, interquartile range 0.01–0.097 events/s). Notably, activity rates of neurons in cultured slices were close to those under an awake *in vivo* condition^25^. Second, the activity of pairs of neurons was only weakly correlated (Fig. 1D). Average correlation coefficient was 0.033 ± 0.065 SD (detection in a 100 ms window). A cross-correlogram revealed that, on average, the activity of pairs of neurons was not correlated after a ~ 400 ms timelapse (Fig. 1D inset). Third, intracellular voltage recordings under the same experimental conditions all reveal uni-modal distributions of membrane potentials (Fig. 1E). Hence, no obvious sign of a superposition of UP and DOWN states was detected.

To analyze the correlated activity of multiple neurons, 50 groups of 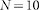 neighboring neurons were selected from each of 20 slices (see an example group of neurons shaded in pink in Fig. 1B and events marked in red in Fig. 1A), for a total of 1000 groups of 10 nearest-neighbor cells. The centers of groups were sampled according to the spatial density of cells in the CA3 area (See Methods). The average ‘radius’ of the 1000 groups was 36.6 (±13.4 SD) μm, where the radius of a group was computed as the mean Euclidean distance of its cell positions from the group's center position. We then represented the activity of the *i*th neuron 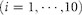 in a time window by a binary variable 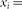
{0,1}, where ‘1’ denotes an active state in which at least one event occurred, and ‘0’ represents an inactive, or ‘silent’, state in which no events occurred (Fig. 1F). We used a 400 ms time-window in the subsequent analyses to incorporate the temporal correlation observed in the cross-correlogram (c.f. the shaded interval in Fig. 1D inset).

**Figure 2:**
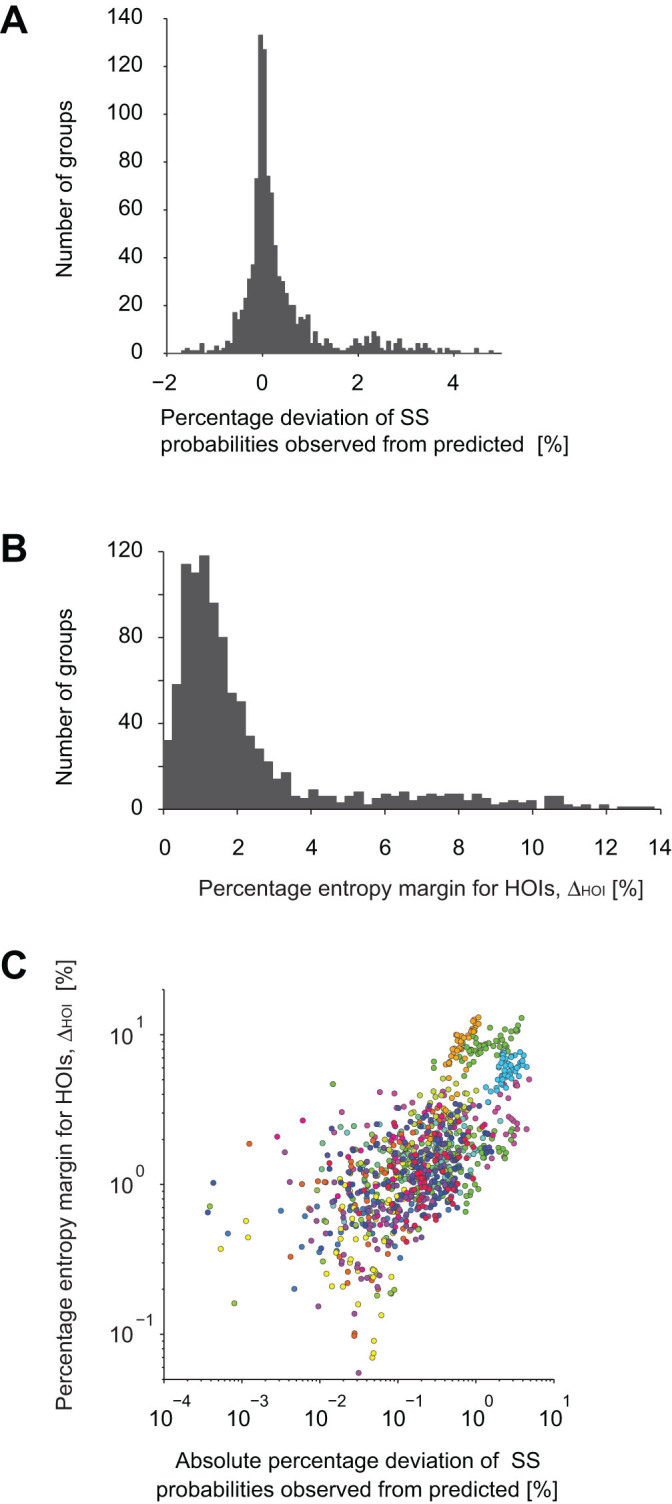
Sub-groups of 10 hippocampal neurons exhibit longer periods of SS than predicted from pairwise interactions. (A) Distribution of the percentage deviation of the observed probability of SS of 10 neurons from the prediction of the pairwise model, 
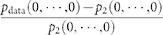
. Positive values indicate more frequent SS in the data than predicted by the pairwise model. (B) Histogram of percentage entropy margins for HOIs computed as 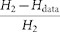. (C) Dependency of the percentage entropy margin for HOIs on the percentage deviation of the observed from predicted probabilities of SS. The same color indicates groups selected from the same slice culture. 5 outliers were excluded from the plots.

To examine if hippocampal neurons exhibit collective activity beyond what can be explained by pairwise interactions, we compared the activity patterns of a group of observed neurons with those predicted from a pairwise maximum entropy model^8^,^9^,^31^, 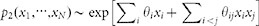. This model provides the least structured probability distribution that is consistent with the observed activity rates of individual neurons and correlations between pairs of neurons. The parameters 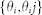 were adjusted to fit these statistics. We call this model a pairwise model hereafter. First, we examined if the neurons exhibited SS beyond that predicted by the pairwise correlations. To this end, we compared the observed probability of the pattern in which all of 10 neurons are simultaneously silent with its probability according to the pairwise model. Figure 2A displays a distribution of percentage deviation of observed SS probabilities from the prediction of the pairwise model, 
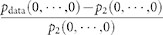
, where 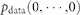 is the observed probability of SS. In some groups, the pairwise model tended to underestimate the occurrence probability of SS of 10 neurons. This discrepancy has to be explained by HOIs in the data.

To examine the contribution of HOIs to population activity, we computed the fraction of entropy that is explained by HOIs. This fraction, referred to as the percentage entropy margin for HOIs, is quantified as 
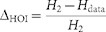
, where 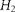 is the entropy of the pairwise model and 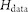 is the entropy of the observed histogram of population activity patterns. We call 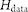 the data entropy in the following. The data entropy is characterized by all of the first, second, and HOIs. Therefore, the difference between 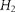 and 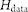 must be explained by HOIs. We found that the distribution of 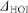 exhibited a long tail (Fig. 2B). This indicates that there were a noticeable number of groups in which HOIs played a much stronger role in shaping population activity. Finally, we explored the relation between the contributions of HOIs to the probability of SS. We found that the groups expressing higher/lower probabilities of SS than the pairwise model coincided with the groups possessing large entropy margins for HOIs (Fig. 2C). The positive correlation between these two values in Fig. 2C (Spearman's rank correlation coefficient 0.69, p <0.001) implies that a significant portion of the HOIs of the CA3 neurons may be explained by the SS. The rank correlation coefficient was higher (0.92) and statistically significant (*p* < 0.001) if we analyze non-overlapping groups.

### Simultaneous silence is a ubiquitous feature of HOIs

To directly examine the contribution of the SS to the entropy explained by HOIs, we constructed a maximum entropy model that augments the pairwise model with a single additional term to account for the probability of SS observed in the data. We refer to this model as the SS model:
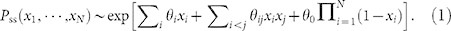
Here a single parameter, 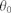, was introduced to account for the probability of SS of 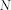 neurons. Positive or negative 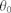 indicates that the probability of SS of all neurons is more or less than predicted by the pairwise model, respectively. Importantly, this new SS term is equivalent to adding specific structured HOIs into the pairwise model. By expanding the SS term into the standard HOI-coordinates, we obtain 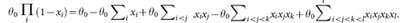 Hence, increasing or decreasing the total period of quiescence is equivalent to introducing a single parameter to the HOIs with alternating signs for different orders of interaction. In addition to capturing individual activity rates and pairwise correlations, the SS model explores this 1-dimensional structure in the high-dimensional space of HOIs to fit the rate of SS. We fitted the SS model to the same 1000 groups of 10 hippocampal neurons obtained from 20 slices. Note that, in each group, the fitted first and second order parameters of the SS model are generally different from those of the pairwise model because of the newly introduced SS term.

**Figure 3:**
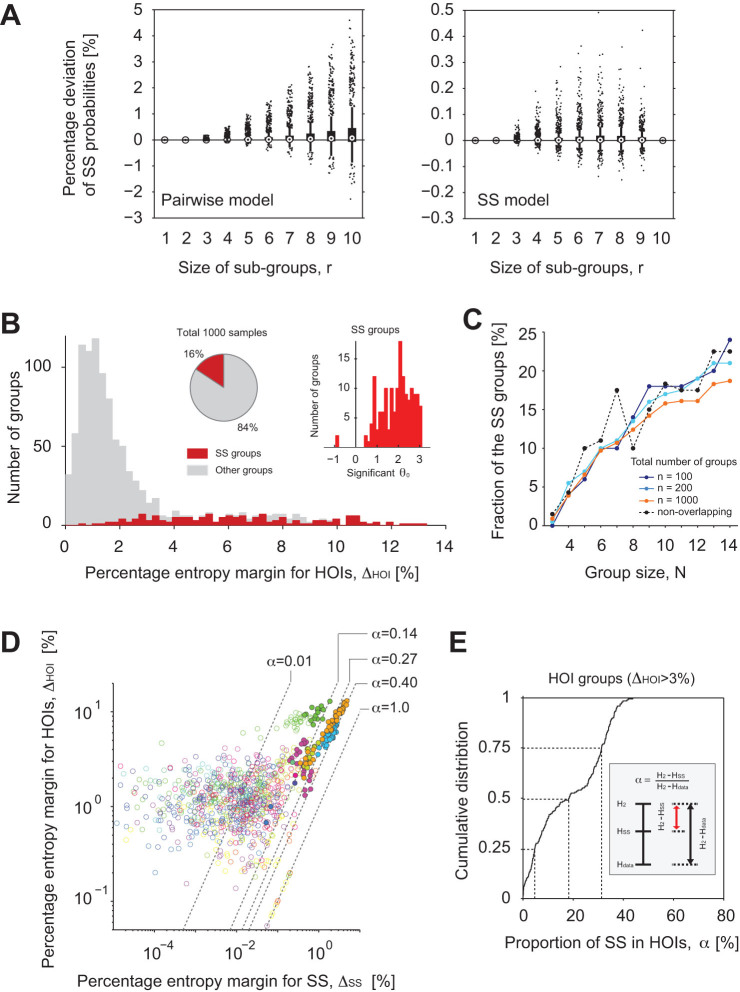
Significant SS is observed in the groups of 10 neurons exhibiting HOIs. (A) (Left) Comparison of the observed SS probabilities of sub-groups of 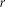 neurons with predictions of the pairwise model. Abscissa, the size 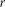 of sub-groups. Ordinate, the percentage deviation of observed from predicted average SS probabilities of sub-groups of neurons, where the normalization divides the difference by SS probability predicted from a pairwise model. The comparison was performed for all possible sub-groups of 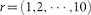 neurons in the 1000 groups of 10 neurons. Whiskers represent 1.5 times the distance from 25th to 75th percentile. Dots are outliers. (Right) Comparison of the observed SS probabilities of the sub-groups with predictions of the SS model. Note the difference in the scales of the ordinates in the Left and Right panels. (B) Distribution of percentage entropy margins for HOIs for the SS groups (red, 16%), i.e. groups for which the SS model showed significantly better fit than the pairwise model, and the rest of the groups (gray, 84%). The right inset shows distribution of the parameter 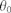 of the SS groups. The distribution was heavily biased toward positive 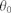, indicating prevalent excess SS. (C) Group size dependency of the number of SS groups. Solid lines with different colors indicate different numbers of groups selected from each slice: 5, 10, 50 groups per slice, for a total of 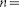
100, 200, and 1000 groups of size 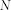 from 20 slices, respectively. The dashed black line is the result of selecting non-overlapping groups from each slice (see Methods). Thus, the fraction of SS groups and its group size dependency were robust to the degree of overlap between sampled groups in each slice. (D) Scatter plots of entropy margins for HOIs versus entropy margins for SS. The same color indicates groups selected from the same slice culture. Filled circles indicate SS groups. Dashed lines represent different proportions of HOI entropy margin explained by the SS term, 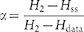. We excluded 13 outliers from the plots. (E) Cumulative distribution function of the proportion of SS, 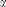, in the groups exhibiting HOIs (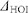
> 3%).

We compared goodness-of-fit of the SS model with that of the pairwise model (Fig. 3A). The ordinate of the panels represents percentage differences between observed and predicted SS probabilities of sub-groups of 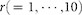 neurons by the two models (Left, the pairwise model; Right, the SS model). By definition, the pairwise model adjusts the silence rates of individual neurons (equivalent to 1 minus activity rates, 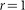) and pairs (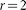) (Fig. 3A Left panel). However, the pairwise model fitted to the data underestimated probabilities of SS for larger sub-groups of neurons. This means that many sub-groups of hippocampal neurons expressed SS more often than chance as predicted from their activity rates and the pairwise correlations. In contrast, the SS model additionally accounts for the probability of SS of all 10 neurons in a group (see the complete match of the data and prediction at 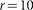 in addition to 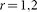 in Fig. 3A Right panel). Order of magnitude reductions in the differences were observed in the SS of many sub-groups (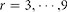). (Note the scale difference in the Left and Right panels.).

We tested the excess or paucity of SS using the SS model against a null hypothesis of no such activity (i.e., the hypothesis that the pairwise model is sufficient to characterize the data). Here, we used 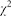-tests^11^ with multiple comparison correction using the Benjamini-Hochberg-Yekutieli method with a false discovery rate of 0.05 to assess if the SS term significantly improved the fitting in each group (See Methods). Of 1000 groups, 156 groups (16%) from 10 slices rejected the null hypothesis (Fig. 3B). We call these groups that exhibit excess or paucity of SS the SS groups. Statistical properties of the SS as well as non-SS groups were summarized in Table 1. Indeed, most of the groups (68%, 133 groups out of 197) that exhibited relatively large margins of entropy for HOIs (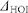
> 3%) were the SS groups. Note that, at this point, each of the SS groups could have had either significantly positive or negative 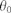. It turned out that 154 out of the 156 SS groups exhibited significantly positive 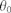 (Fig. 3B Right inset). Thus, virtually all the SS groups expressed significantly larger probability of SS than the corresponding pairwise model. In these groups, the total number of bins for which all neurons were quiet was larger than expected by the corresponding pairwise model. In other words, activity was confined to a smaller number of bins. Hence, we conclude that the population activity of most groups exhibiting HOIs (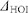
> 3%) was significantly sparse in time. Note that the observed fraction of SS groups was robust to the number of groups sampled from each slice but typically increased with the size of these groups (Fig. 3C).

Finally, we examined the relation between the percentage entropy margin for HOIs, 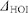, and the percentage of the HOI entropy explained by the SS. The latter entropy was computed as 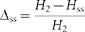, where 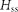 is the entropy of the SS model. Figure 3D displays scatter plots of these values for all groups. As predicted from Fig. 2C, we observe significant positive correlation between 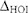 and 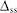 (Spearman's rank correlation coefficient 0.52, p <0.001). The dashed lines are isoclines of a constant ratio 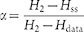. This ratio describes the fraction of entropy explained by the SS in the entropy margin for HOIs. As expected, the SS groups (filled circles) typically had large 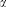. Figure 3E displays a distribution of 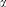 for the groups that expressed large margin for HOIs (197 groups with 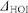
> 3%). In these groups, the single higher-order parameter of the SS explained 18.3% (interquartile range, 4.7–31%) of the entropy for HOIs (Fig. 3C). Since we have only added a single parameter in the high-dimensional space of HOIs, this result implies that the SS comprises one important characteristic of the HOIs.

In order to assess biases that may be caused by limited samples in our data sets, we repeated our analysis using two alternative data sets (Supplementary Fig. S1 online). First, we analyzed only one half of the data by taking every other bin of the original population activity patterns for each slice. Second, we analyzed bootstrapped population activity patterns, where the same number of patterns as the original data were resampled with replacement in each slice. These two data sets contain less variations of population activity patterns than the original data. For the both data sets, the fraction of SS groups was smaller than the 16% found in Fig. 3B. The fraction of the HOIs explained by SS also decreased to less than a half of 18% found in Fig. 3E. Because we did not overestimate these quantities after subsampling and resampling, it is unlikely that our original estimation (16% exhibits significant SS; 18% of HOIs is explained by SS) overestimated the fractions expected from a larger number of samples. In sum, the analyses confirm significant SS in the data, and predict the presence of the alternating signs of HOIs, a possibility we directly test now.

### Alternating signs of HOIs predicted by SS

If SS is a major feature of the HOIs, we expect to find HOIs whose signs alternate depending on the orders of interaction (c.f. the expansion of the SS term). In order to directly examine the structure of HOIs, we consider a simple maximum entropy model that includes a single global parameter for each order of HOIs:
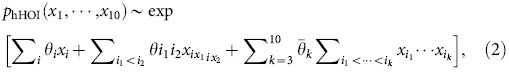
where 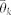 (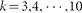) is a single parameter for the 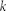
th order HOIs. The term for the 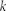
th order interaction parameterized by a parameter 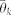 sums all combinatorial interactions of 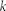 neurons among 10 neurons. We call this model the homogeneous HOI (hHOI) model. The hHOI model fitted to the data reproduces the histogram of the number of active neurons in each time bin^14^,^19^,^20^,^32^.

**Figure 4:**
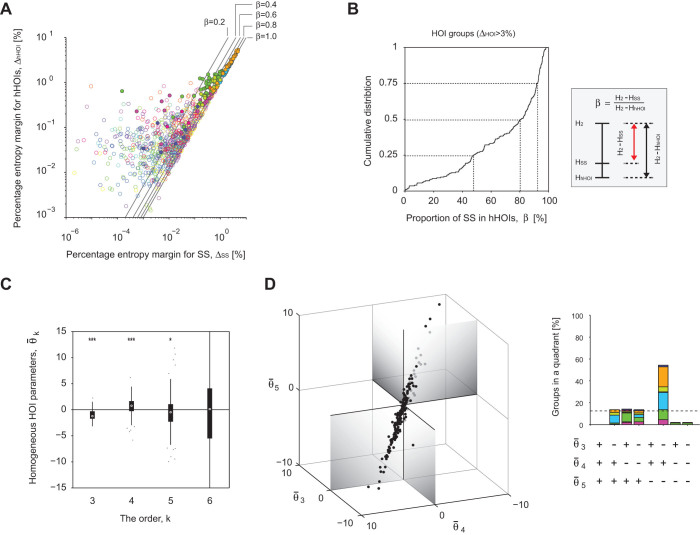
The groups that express HOIs exhibited alternating signs of homogenous HOIs at successive orders of interaction. (A) Scatter plots of entropy margins for hHOIs versus entropy margins for SS. The same color indicates groups selected from the same slice culture. Filled circles indicate groups exhibiting HOIs (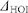
> 3%). Dashed lines represent different proportions of homogenous HOI entropy margin explained by the SS term, 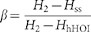. We excluded 9 outliers from the plots. (B) Cumulative distribution function of this proportion, 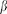, for the groups exhibiting HOIs (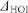
> 3%). (C) The homogeneous HOI parameters up to the 6th order of the hHOI model. Each box covers 25th to 75th percentile, and whiskers represent 1.5 times the distance from the 25th to 75th percentile. Dots are outliers. The distributions at the 3rd, 4th, and 5th order deviated significantly from zero (two tailed sign test, *** and * represent significance level 0.001 and 0.05, respectively). (D) (Left) 3-dimensional plots of the homogeneous HOIs of the hHOI model. Outliers with elements larger than 10 or less than −10 were excluded. (Right) A histogram of the number of groups that fell in 8 quadrants of the 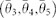 parameter space. The same color marks the same slice culture. The dotted horizontal line is the chance level (12.5%) with random HOIs.

In these data sets (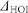
> 3%), the hHOI model explained 24% of the variability in population activity due to HOIs (interquartile range 11–34%) as assessed by 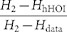, where 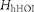 is the entropy of the hHOI model. This result indicates prevalent heterogeneity in the HOIs. The result also upper bounds the fraction of entropy for HOIs that could be explained by the single SS term. We then investigated how much of this entropy is actually explained by the SS term. Figure 4A displays relations between the percentage entropy margin explained by the hHOI model, 
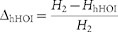
, and by the SS model, 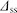. Similarly to Fig. 3D, the dashed lines are isoclines of 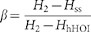, which quantifies the fraction of entropy explained by the SS in the entropy margin for the homogenous HOIs. Figure 4B shows a distribution of 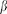 for the groups exhibiting HOIs (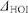
> 3%). In these groups, the single SS term explained 80% of the entropy for homogeneous HOIs (interquartile range 48–92%). From this result, we conclude that SS constitutes the dominant structure of the homogeneous HOIs.

We next directly visualize the structure of homogeneous HOIs. Figure 4C displays distributions of the homogeneous HOI parameters, 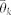 of the hHOI models. (We only show the parameters for 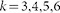 although we fitted hHOIs up to the 10th order). The homogeneous HOI parameters up to the fifth order but not higher were significantly different from zero (two tailed sign test). The set of the homogeneous HOI parameters, 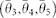, from each group fell in a particular quadrant in the 3-dimensional space (negative triple-wise, positive quadruple-wise, and negative quintuple-wise interactions, Fig. 4D), exhibiting an obviously biased direction. (If the set of homogeneous HOI parameters randomly fell in any quadrant, the probability that the observed number of groups (~ 54% of the groups) would fall in any single quadrant would be less than 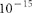). Thus the structured homogeneous HOIs found up to the 5th order contributed to the excess SS found in 68% of the groups with 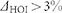. These results demonstrate that the structured HOIs with alternating signs are an attribute of excess SS in local networks of hippocampal neurons.

### Simultaneous silence relies on network inhibition

**Figure 5:**
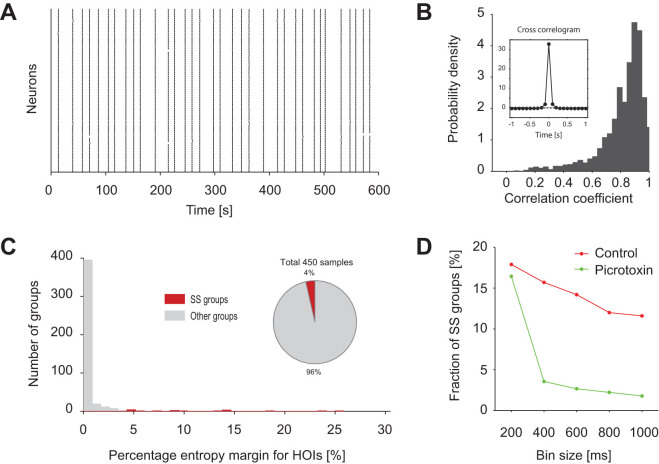
Blocking inhibitory networks by PTX eliminated HOIs. The panels retain the same presentation format as in Fig. 1A, D and Fig. 3B. (A) Ensemble activity of 76 neurons in the CA3 area of a single hippocampal slice under bath application of PTX. (B) Distribution of correlation coefficients between two event sequences (resolution of 100 ms) of all the pairs of neurons in 450 groups from 9 slices. Inset shows an average cross-correlogram. (C) Distribution of percentage entropy margin for HOIs. The groups that showed improved fitting with the SS term are marked in red (the SS groups, 4%). Others (96%) are in gray. (D) The number of the SS groups with respect to the bin size for the analysis.

**Figure 6:**
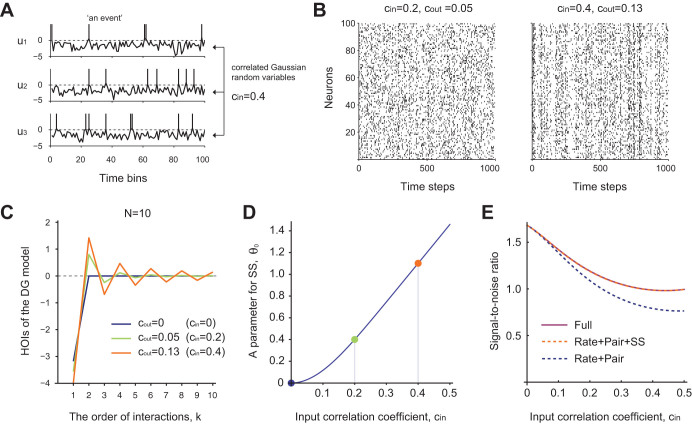
The ensemble activity simulated by the Dichotomized Gaussian (DG) model exhibits alternating signs of HOIs depending on successive orders of interaction. (A) Illustration of a DG model of 3 neurons. The traces in each panel represent correlated input variables, 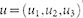 at different time steps. The inputs are sampled from a multivariate Gaussian distribution, 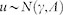, with mean vector 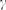 and covariance 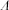 (See Methods). Here we assume that the mean vector contains the same scalar element, 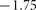, in order to yield the activity probability 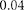, a value close to the empirically observed average activity rate (0.039 per 400 ms window). The off-diagonal elements of 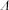 are all fixed at 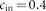. The vertical lines above 0 in each panel mark the time steps at which an input crosses the threshold. (B) Simulation of the DG model with 100 neurons with weak (Left) and strong (Right) input correlations. The weak input correlation (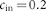) in the Left panel yields a weak correlation coefficient (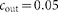) of output binary variables, whereas the strong input correlation (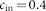) in the Right panel yields a stronger correlation coefficient (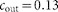) of output binary variables. (C) The HOIs of a small population 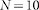 from the DG model shows clear alternation of signs as the order of interaction 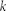 increases except for 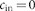. Negative interactions occur at odd 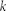 and positive interactions occur at even 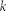. (D) The parameter of SS, 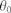, as a function of input correlation coefficient, 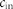. The dots marked in color represent 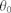 at 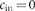, 0.2, and 0.4. (E) The signal-to-noise ratio of the input correlation coefficient, 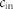, as a function of 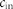 in the population activity of the DG model (The solid purple line). The dotted lines are signal-to-ratios in the subset features of the population activity (blue: the activity rates of individual and pairwise neurons; orange: the activity rates of individual and pairwise neurons plus the SS rate).

Several different biological mechanisms may underlie the observed structure of HOIs. One such mechanism may be the inhibitory networks in the hippocampal CA3 area. To test this hypothesis, we examined population activity under bath application of GABA_A_ receptor antagonist picrotoxin (PTX) (Fig. 5A). When fast GABA_A_ mediated inhibitory networks were blocked by PTX, activities of observed neurons nearly completely synchronized with each other (Fig. 5B). The cross-correlogram exhibited a sharper peak (Fig. 5B inset) than that in the control (cf. Fig. 1C), much shorter than the 400 ms time window used to analyze the control condition. Nonetheless, we used the same window-size, 400 ms, to test for the deviation of SS from the pairwise model, except in Fig. 6D, where we explored the dependency on bin sizes. Table 1 summarizes activity rates, correlation coefficients, and probabilities of SS computed using the 400 ms bin under control and PTX conditions. The average probability of SS under the PTX conditions was much larger than that under the control condition. However, this frequent SS is expected from the high pairwise correlation coefficients observed under the PTX conditions. Indeed, the entropy explained by HOIs was greatly diminished in the PTX data, indicating that the pairwise model adequately explained population activity in almost all groups under blockade of inhibition (Fig. 5C). Accordingly, the percentage of groups that exhibited significant SS beyond the pairwise model was considerably reduced from 16% down to 4% (Fig. 5C, red). The considerable reduction of SS groups was observed whenever the window size larger than 200 ms was used in order to thoroughly cover the synchronous events (Fig. 5D). We thus concluded that an inhibitory network is necessary for neurons to produce both frequent SS and weak pairwise correlations; the conjunction of both can only be explained by HOIs.

### Simultaneous silence emerges in a population of thresholding units that receive correlated input

Finally, we demonstrated that a simple model of neural population reproduces the structured HOIs with alternating signs with respect to different orders of interaction observed in the spontaneous activity of hippocampal neurons under the control conditions. A population model known as the Dichotomized Gaussian (DG) model^33^,^34^,^35^,^36^ simulates a population of neurons that receive correlated Gaussian inputs, where each neuron produces a binary output in response to its input by simple thresholding (Fig. 6A, See Methods). Despite the substantial simplification, the spiking mechanism is similar to the one assumed in networks of balanced excitatory and inhibitory neurons: the mean input to each neuron is typically smaller than the threshold and, therefore, spikes are induced by fluctuations in the input. The DG model has been reported to reproduce neural activity patterns better than the pairwise model^22^. Figure 6B displays simulated DG models using different strengths of input correlations (Fig. 6B), including one that produces output correlations similar to those found in experimental observations (see Table 1). The population exhibited asynchronous spiking activity. We numerically computed the HOIs of the DG model for 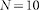 (See Methods). The HOIs showed clear alternation in signs with respect to the successive orders of interaction (Fig. 6C) and demonstrated excess SS (Fig. 6D). These results show that the experimentally observed SS with structured HOIs can arise from the conjunction of two ubiquitous biological features, i.e., correlated input and spiking nonlinearity. Further, we demonstrate that SS can contain rich information of inputs provided to the observed population of neurons. Figure 6E compares the signal-to-noise ratio for estimating the input correlation based on specific features of population activities – activity rates and pairwise correlations (Rate + Pair), SS in addition to activity rates and correlations (Rate + Pair + SS), and joint activity rates of all orders (Full). The signal-to-noise ratio, which is also called the linear Fisher information^37^,^38^,^39^, quantifies the accuracy of estimating a small change in an input parameter by an optimal liner decoder (see Methods). The result shows that measuring SS in addition to activity rates and correlations can provide nearly full information available from the observation of all statistics. The results were qualitatively the same when an input mean (or a threshold level) was estimated instead of input correlation.

## Discussion

We investigated the structure of HOIs in spontaneous activity of neurons in the CA3 area of organotypic hippocampal slice cultures. Most groups (~ 70%) of neurons that expressed significant HOIs (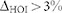) also exhibited excess SS (Fig. 3), and SS alone could account for ~ 20% of the entropy explained by HOIs in these groups. This result predicts significantly biased homogeneous HOIs with alternating signs at successive orders of interaction and our data analysis confirmed this prediction (Fig. 4). We also found that SS explained 80% of the entropy due to structured homogeneous HOIs.

SS was robustly observed across a range of time-bins (Fig. 5D) and sizes of neural populations (Fig. 3C). Moreover, SS and the resulting structure of HOIs arise in the simplest model of a neural population that possesses a spiking nonlinearity and correlated inputs (the DG model), where additional observation of SS is sufficient to decode most information of input conveyed by different orders of HOIs. These results suggest that excess SS is an important and ubiquitous characteristic of neural population activity that summarizes its low-dimensional structure in the combinatorial space of HOIs. We identified alternating signs of HOIs up to the 5th order with statistical significance in the analyzed data (Fig. 4C and D), and the DG model displayed the predicted structure up to the highest order of interaction (Fig. 6C). Based on these observations, we speculate that the predicted structure of HOIs beyond the 5th order should be identifiable in future, given longer experimental recordings. We also speculate that appropriate models^5^,^15^,^21^ of neural population activity implicitly include excess SS as well as the resulting structure of HOIs, as demonstrated in the DG model.

Multiple biological mechanisms may underlie the high SS probability observed and alternating signs of HOIs. While we have shown that even simple thresholding units with correlated inputs can reproduce this structure, we do not exclude contributions from other mechanisms. Indeed, we demonstrated the involvement of inhibitory input in generating SS (Fig. 5). Under the blockade of GABA_A_ receptors, activities of neurons were almost completely synchronized. Therefore HOIs of the population activity were significantly diminished. It is expected that neurons are almost fully synchronized and fire regularly if inhibition is removed^40^. However, it may require additional neuronal mechanisms with slow dynamics^41^,^42^ to robustly account for the sparse synchronous activity observed in the current data sets. While this result may simply indicate that inhibition is necessary to place a network of neurons in a fluctuation-driven regime^43^,^44^ for them to be sensitive to correlated input (c.f. Fig. 6), it may alternatively suggest the existence of clustered inhibitory input that simultaneously shuts down a group of local neurons and produces excess SS. Inhibitory interneurons in the hippocampus have diverging connections to principal neurons^45^ and show powerful control over timing and rhythms of their spiking activity^46^,^47^. Such inhibitory circuits are ideally suited to implement a winner-take-all-like competition among groups of neurons, which are common in models of hippocampal circuits aiming to reproduce place fields^48^,^49^. Similar operations of hippocampal inhibitory circuits have also been suggested for cellular assemblies^50^ and memory consolidation^51^. Thus the excess SS in spontaneous activity reported here might be related to functions requiring sparse information representation with a small fraction of active neurons. It is therefore interesting to see if the same experimental manipulation of inhibition that influences, for example, the sparse place field representation, also influences SS during spontaneous activity.

Our study demonstrates that excess SS explains a large fraction of the variability caused by complex HOIs in neural populations. Although it was previously reported that HOIs fitted to several representative activity patterns explain occurrence probabilities of other general patterns^18^, this study did not normalize the model probability distribution because of the computational complexity associated with the normalization step. As a drawback, it was previously unknown how much of the variability associated with HOIs was explained by a small number of representative activity patterns. In contrast, the entropy maximization approach we have taken was suitable to evaluate these quantities. More generally, virtually all previous studies of HOIs^14^,^19^,^20^ attempted to fit multiple model parameters to the data rather than to extract the most prominent feature in the space of HOIs. Note that the hHOI model fitted to the data reproduces an observed histogram of the number of active neurons in time bins (i.e., a population spike-count histogram). Thus the hHOI model is equivalent to the K-pairwise model proposed in Tkačik et al.^20^ although the two models utilize different features of activity patterns to represent homogenous HOIs. We have found that SS can parsimoniously summarize 80% of the specific structure of hHOIs. Furthermore, successive orders of interaction have alternating signs. This resulting structure extends the negative triple-wise interactions previously found in local (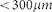) populations of 3 neurons^17^.

In sum, we demonstrate that representing HOIs using “silence” provides a much more concise description than the canonical representation based on “activity”. We conclude that significant SS is a ubiquitous feature in neural population activity that expresses apparently diverse HOIs across different orders.

## Methods

### Recording method

Hippocampal slice cultures were prepared from postnatal day 7 Wistar/ST rats (SLC) (either male or female). Entorhino-hippocampal stumps were cultivated on membrane filters using 50% minimal essential medium, 25% Hanks' balanced salt solution, 25% horse serum, and antibiotics in a humidified incubator at 37°C in 5% CO_2_ and were used for experiments on days 7 to 14 *in vitro*. On experimental days, slices were washed with oxygenated artificial cerebrospinal fluid (aCSF) consisting of (mM) 127 NaCl, 26 NaHCO_3_, 3.3 KCl, 1.24 KH_2_PO_4_, 1.2 MgSO_4_, 1.2 CaCl_2_, and 10 glucose and bubbled with 95% O_2_ and 5% CO_2_. They were then transferred to a 35-mm dish filled with 2 ml of dye solution and incubated for 40 min in a humidified incubator at 37°C in 5% CO_2_ with 0.0005% Oregon Green 488 BAPTA-1 (OGB-1) AM (Invitrogen), 0.01% Pluronic F-127 (Invitrogen), and 0.005% Cremophor EL (Sigma-Aldrich). They were recovered in aCSF for> 30 min and mounted in a recording chamber at 32°C and perfused with aCSF at a rate of 1.5–2.0 ml/min for > 15 min. Hippocampal CA3 pyramidal cell layer was imaged at 10 Hz using a Nipkow-disk confocal microscopy (CSU-X1; Yokogawa Electric), a cooled CCD camera (iXonEM^+^ DV897; Andor Technology), an upright microscope with a water-immersion objective lens (16 ×, 0.8 numerical aperture, Nikon). Fluorophores were excited at 488 nm with a laser diode and visualized with a 507-nm long-pass emission filter. The recording lengths varied from 600 sec to 3300 sec (600 sec (*n* = 9); 1200 sec (*n* = 4); 310, 610, 700, 900, 1100, 1800, 3300 s (*n* = 1)). Picrotoxin was bath-applied at a concentration of 50 µM to 9 slices (600 sec (*n* = 7) and 350 s (*n* = 2)). After identification of cell types, the regions of interest (ROIs) were carefully placed onto the cell bodies. The fluorescence change (Δ*F*/*F*) was calculated as Δ*F*/*F* = (*F*_t_–*F*_0_)/*F*_0_, where *F*_t_ is the fluorescence intensity at time *t*, and *F*_0_ is the baseline averaged for 50 s before and after time *t*. For neurons, event times were reconstructed from the onsets of Ca^2+^ transients^3^,^27^,^28^. The signals were then inspected by eye to remove erroneously detected noise. The data is available online (http://gaya.jp/data). Under the same condition as described above, membrane potentials were whole-cell recorded at I = 0 from pyramidal cells (*n* = 7) visually identified under infrared differential interference contrast microscopy. Patch pipettes (3−6 MΩ) were filled with a solution consisting of (in mM) 120 K-gluconate, 10 KCl, 10 HEPES, 10 creatine phosphate, 4 MgATP, 0.3 Na_2_GTP, and 0.2 EGTA. The signal was digitized at 10 kHz and filtered with a band of 1–2000 Hz. Liquid junction potentials were not corrected. Experiments were performed with the approval of the animal experiment ethics committee at the University of Tokyo (approval No. P24-6) and according to the University of Tokyo guidelines for the care and use of laboratory animals. All efforts were made to minimize the animals' suffering and the number of animals used.

### Selection of groups of neighboring neurons

From each slice, we selected 50 distinct overlapping groups, each consisting of 10 nearest-neighbor neurons, based on the following procedure. In each slice, we estimated the density of spatial distribution of the cells in the recorded area of CA3 by an optimized 2-dimensional kernel density estimation method^52^. We then sampled a spatial point from the estimated density, and selected the 10 neurons nearest to the point. We repeated this procedure until we obtained 50 distinct groups (we discarded groups of neurons if the exactly same group of 10 was previously selected). The neurons with low activity rates (less than 0.01 Hz) were excluded from this analysis. In addition, we changed the number of neurons in a group from 3 to 14 to investigate effect of the group size (Fig. 3C). We sampled up to 50 groups per slice following the same sampling procedure described above. Finally, we sampled non-overlapping groups of 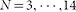 neurons. Note that we can sample only a small number of groups from each slice if groups are stochastically sampled by the above-mentioned method. Thus, we took the following procedure to efficiently select non-overlapping groups from each slice. First, we fitted a 2-dimensional Gaussian density function to the spatial distribution of cells in each slice. We then determined the first principle component, and scored the positions of neurons along the first principle axis. We selected neurons that are nearest neighbors in terms of this score as a group. To determine the number of groups sampled from each slice, we computed the maximum number of non-overlapping groups that can be sampled from each of all slices. We used the smallest number of groups among them to sample an equal number of groups from each slice.

### Model fitting and a test of simultaneous silence

First, we fit to binary population activity data (see Fig. 1F in Results) the pairwise maximum entropy model^8^,^9^,^31^, 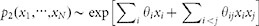, where 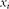 is a binary variable of 0 or 1. Here the parameters of the model, 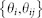, were fitted by a maximum likelihood principle, 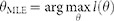, where 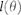 is the log likelihood of the data under the model. The nonlinear fitting was performed using a custom convex optimization program in Matlab. We then fit to the same data a maximum entropy model that augments the pairwise model with a single term to account for the observed probability of SS in addition (the SS model, see Eq. 1 in Results). The increase in likelihood over the pairwise model seen after adding a parameter for the SS is related to the reduction in entropy by 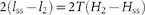, where 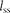 and 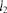 are the log likelihood of the data under the SS model and the pairwise model respectively, and 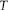 is the number of observed patterns (bins). Under the null hypothesis of no such SS term, the variability in the model estimation due to finite samples make the difference in log likelihood following a 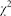-distribution with one degree of freedom as 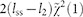
11. The p-value of the observed likelihood increase was computed using this null distribution. The p-values were further corrected by the Benjamini-Hochberg-Yekutieli multiple comparison correction method that is applicable to dependent tests, using Matlab code written by Groppe et al.^53^,^54^ This method controls the proportion of tests that incorrectly declare significant SS (the false discovery rate).

### A dichotomized gaussian (DG) model

The DG model is a threshold neuron model with Gaussian input signals^33^,^34^,^35^,^36^. The binary output of the 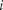-th neuron (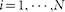) is given by 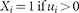 or 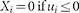, where 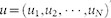 is drawn from a multivariate Gaussian distribution with mean 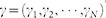 and a covariance matrix 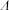 whose diagonal is 1 as 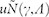. Note that  describes matrix (or vector) transpose. Here we consider a homogenous neuron pool: the mean is all fixed at 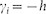 and the off-diagonal elements of 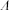 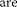 all fixed at 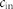. The probability that individual output neurons are in an active state is given by 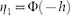, where 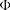 is the one-dimensional cumulative distribution function (CDF) of a zero-mean, unit variance Gaussian distribution. The probability of simultaneous activity of 2 neurons is given by 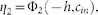 where 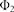 is the 2-dimensional Gaussian CDF with zero-means, unit variances, and a off-diagonal correlation coefficient 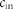. The correlation coefficient between 2 output neurons is given by 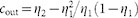.

The probability distribution of population activity has a simple analytical expression in this model. Note that the correlated inputs can be written as 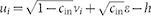, where 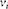 is a unit variance white Gaussian noise 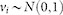 specific to each neuron, and 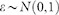 is an input noise that is common across all neurons. The conditional probability of a single neuron spiking given the common input 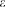 is given by^33^
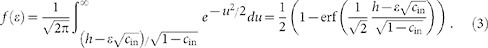
The probability that exactly 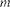 neurons are active and 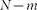 neurons are inactive is given 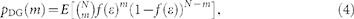
where the expectation is performed with respect to the common input noise, 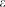. Note that the binomial factor 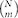 sums all possible combinations of population activity patterns with 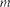 active neurons. In order to obtain the probability mass function for the finite population size 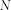, we numerically computed the above equation. On the other hand, the same population-count probabilities are described by
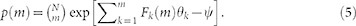
Here 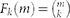 (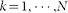) is the *k-*th order feature of the hHOI model, which counts all combinations of choosing *k* neurons out of *m* active neurons, and 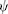 is a normalization factor. Thus, by solving linear equations, 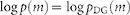 for 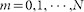, we obtain the parameters, 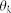 (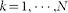), and the normalization factor 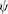.

We quantify the signal-to-noise ratio for estimating the input correlation, 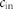, based on the population activity of the homogenous DG model as follows. A small change in 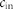 is inferred from a vector of observation, 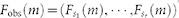, where indices 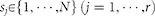 specify a subset of *r* features that are taken into account for the inference. The signal for detecting the input correlation is given by 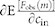 and the noise of the observation is quantified by 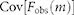, where 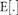 is the expectation and 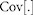 is the 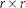 covariance matrix calculated using 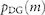 defined above. Together, the signal-to-noise ratio is given by 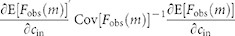
38,39. In the paper, we specifically consider three types of observations as 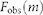: the full observation 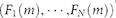, the activity rates of individual and pairwise neurons 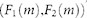, and these activity rates plus the SS rate 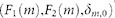, where 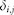 represents Kronecker's delta. Notably, when all the features 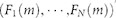 are observed, the above signal-to-noise ratio becomes equivalent to the Fisher information^55^ of the input correlation, i.e., 
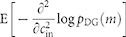
, and thus upper-bounds the accuracy of unbiased estimators of 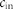 based on the population activity. See^56^,^57^ for information in subset features achieved by a general optimal nonlinear decoder as assessed by the Fisher information.

## Author Contributions

H.S.: Conception and design; Analysis and interpretation of data; Drafting and revising the article. K.S.: Drafting and revising the article. T.I.: Acquisition of data; Revising the article. Y.I.: Acquisition of data; Drafting and revising the article. T.T.: Conception and design; Analysis and interpretation of data; Drafting and revising the article.

## Supplementary Material

Supplementary Information Supplementary Figure S1

## Figures and Tables

**Table 1:** Activity rates, correlation coefficients, and probabilities of SS computed from binary sequences using 400 ms window under control and PTX conditions. Values are expressed as Mean (± SD)

	Activity rate	Correlation coefficient	Prob. SS
Control	0.039 (± 0.042)	0.060 (± 0.108)	0.728 (± 0.152)
SS groups	0.032 (± 0.035)	0.172 (± 0.122)	0.831 (± 0.103)
Non-SS gropus	0.040 (± 0.043)	0.040 (± 0.092)	0.706 (± 0.151)
PTX	0.027 (± 0.017)	0.920 (± 0.108)	0.965 (± 0.021)
