# Erratum: Simultaneous silence organizes structured higher-order interactions in neural populations

**DOI:** 10.1038/srep14893

**Published:** 2015-10-09

**Authors:** Hideaki Shimazaki, Kolia Sadeghi, Tomoe Ishikawa, Yuji Ikegaya, Taro Toyoizumi

Scientific Reports 5: Article number: 982110.1038/srep09821; published online: 04282015; updated: 10092015

This Article contains typographical errors.

In the Results section under subheading ‘Simultaneous silence and HOIs of hippocampal neurons’

“The parameters *{θ*_*i*,_*θ*_*i*_*j}* were adjusted to fit these statistics.”

should read:

“The parameters *{θ*_*i*,_*θ*_*ij*_*}* were adjusted to fit these statistics.”

In the Results section under subheading ‘Simultaneous silence is a ubiquitous feature of HOIs’

“By expanding the SS term into the standard HOI-coordinates, we obtain


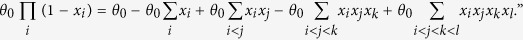


should read:

“By expanding the SS term into the standard HOI-coordinates, we obtain


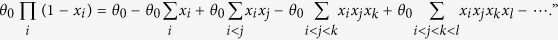


In the Result section under subheading ‘Alternating signs of HOIs predicted by SS’, equation (2)


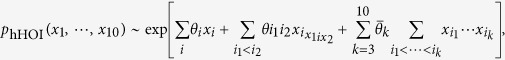


should read:


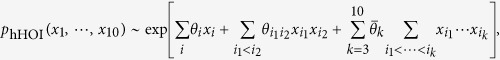


In the Methods section under subheading ‘A dichotomized gaussian (DG) model’,

“The binary output of the *i*-th neuron (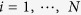) is given by 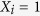 if 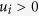 or 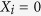 if 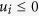, where 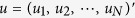 is drawn from a multivariate Gaussian distribution with mean 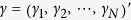 and a covariance matrix 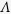 whose diagonal is 1 as 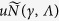.”

should read:

“The binary output of the *i*-th neuron (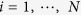) is given by 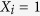 if 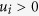 or 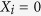 if 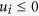, where 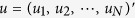 is drawn from a multivariate Gaussian distribution with mean 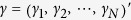 and a covariance matrix 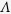 whose diagonal is 1 as 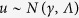.”

In addition,

“The correlation coefficient between 2 output neurons is given by 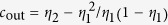.”

should read:

“The correlation coefficient between 2 output neurons is given by 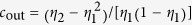.”

In addition,

“The probability that exactly *m* neurons are active and *N*-*m* neurons are inactive is given”

should read:

“The probability that exactly *m* neurons are active and *N*-*m* neurons are inactive is given by”

In addition,

“The signal for detecting the input correlation is given by 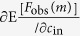 and”

should read:

“The signal for detecting the input correlation is given by 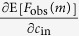 and”

