# Supplementary material for: Simultaneous silence organizes structured higher-order interactions in neural populations
Source: Sci Rep. 2015 Apr 28;5:9821. doi: 10.1038/srep09821 (PMC4412118; doi:10.1038/srep09821)
Supplement: Supplementary Information — Supplementary Figure S1 [file srep09821-s1.pdf]

Simultaneous silence organizes structured higher-order interactions in neural populations

Hideaki Shimazaki, Kolia Sadeghi, Tomoe Ishikawa, Yuji Ikegaya, and Taro Toyoizumi

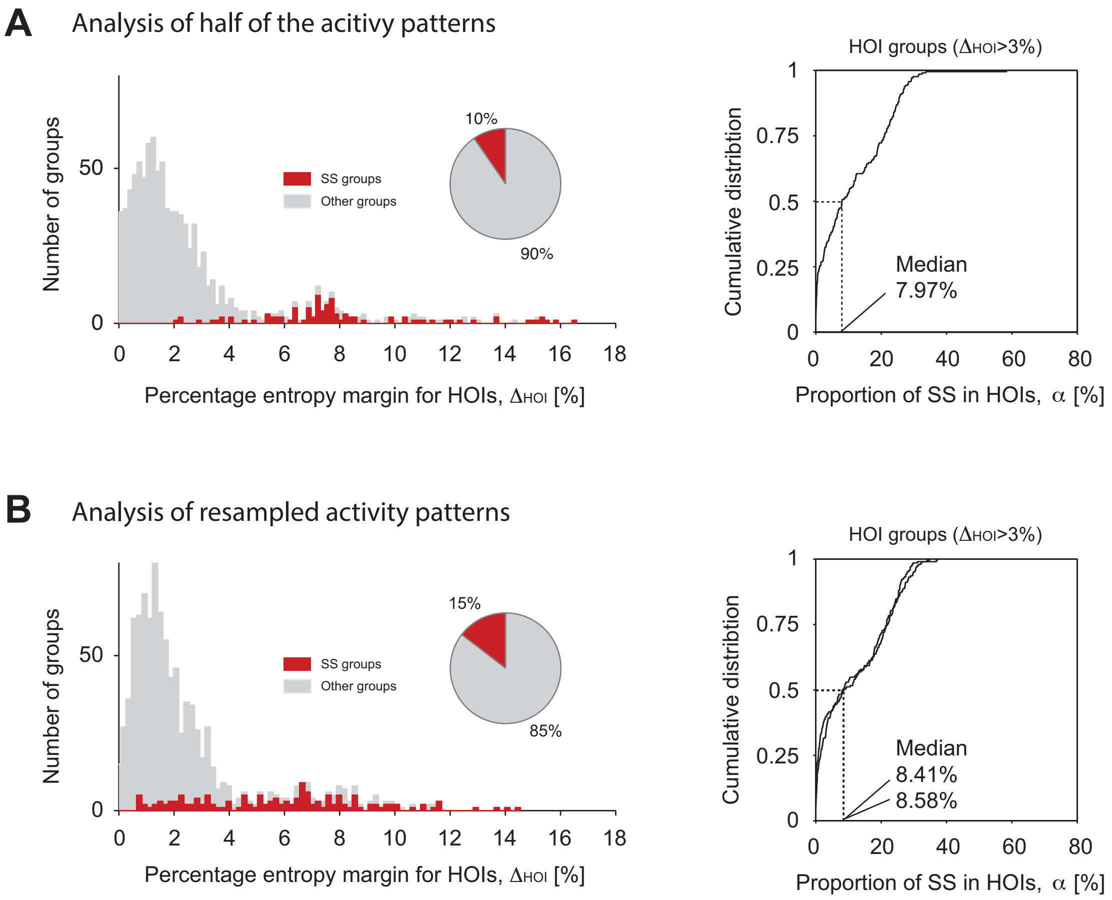

Supplementary Fig. S1 Subsampling or resampling does not lead to overestimation of SS. Conventions as in Fig. 3B, E. (A) Data was constructed by collecting only one half of the data by taking every other bin of the original population activity patterns for each slice. (Left) A distribution of percentage entropy margins for HOIs for the SS groups (red, 10%) and the rest of the groups (gray, 90%). (Right) A cumulative distribution function of the proportion of SS in HOIs (α) of the groups with high HOIs (Δ_{HOI}>3%). The median and an inter quartile range (IQR) are 7.97% and 1.14%-20.97%. (B) The same as in A but with data resampled from the patterns observed in each slice. The same number of patterns as in the original data were resampled with replacement. (Left) A distribution of percentage entropy margins for HOIs. Significant SS were observed in 15% of the groups. (Right) Cumulative distribution functions of α computed from two resampled data sets. The medians are 8.41% (IQR, 0.79%-21.65%) and 8.58% (IQR, 1.46%-21.86%).
